# Efficacy of Plasma-Polymerized Allylamine Coating of Zirconia after Five Years

**DOI:** 10.3390/jcm9092776

**Published:** 2020-08-27

**Authors:** Nadja Rohr, Katja Fricke, Claudia Bergemann, J Barbara Nebe, Jens Fischer

**Affiliations:** 1 Biomaterials and Technology, Department of Reconstructive Dentistry, University Center for Dental Medicine, University of Basel, 4058 Basel, Switzerland; jens.fischer@unibas.ch; 2 Department of Cell Biology, Rostock University Medical Center, 18057 Rostock, Germany; claudia.bergemann@med.uni-rostock.de (C.B.); barbara.nebe@med.uni-rostock.de (J.B.N.); 3 Leibniz Institute for Plasma Science and Technology e.V. (INP), 17489 Greifswald, Germany; k.fricke@inp-greifswald.de

**Keywords:** zirconia implant, human osteoblasts, cell viability, cell spreading, gene expression, plasma-polymerized allylamine, X-ray photoelectron spectroscopy

## Abstract

Plasma-polymerized allylamine (PPAAm) coatings of titanium enhance the cell behavior of osteoblasts. The purpose of the present study was to evaluate a PPAAm nanolayer on zirconia after a storage period of 5 years. Zirconia specimens were directly coated with PPAAm (ZA0) or stored in aseptic packages at room temperature for 5 years (ZA5). Uncoated zirconia specimens (Zmt) and the micro-structured endosseous surface of a zirconia implant (Z14) served as controls. The elemental compositions of the PPAAm coatings were characterized and the viability, spreading and gene expression of human osteoblastic cells (MG-63) were assessed. The presence of amino groups in the PPAAm layer was significantly decreased after 5 years due to oxidation processes. Cell viability after 24 h was significantly higher on uncoated specimens (Zmt) than on all other surfaces. Cell spreading after 20 min was significantly higher for Zmt = ZA0 > ZA5 > Z14, while, after 24 h, spreading also varied significantly between Zmt > ZA0 > ZA5 > Z14. The expression of the mRNA differentiation markers collagen I and osteocalcin was upregulated on untreated surfaces Z14 and Zmt when compared to the PPAAm specimens. Due to the high biocompatibility of zirconia itself, a PPAAm coating may not additionally improve cell behavior.

## 1. Introduction

To replace missing teeth, dental implants made of titanium are a valuable treatment option. However, in recent years, titanium implants have been critically discussed regarding the release of titanium particles and biologic complications [1]. There are some indications that Ti ions released from the implant surface upregulate the expression of chemokines and cytokines in human osteoclasts and osteoblasts. Consequently, osteoclastogenesis is induced, which may contribute to the pathomechanism of aseptic loosening [2,3]. Dental implants made of zirconia can be considered promising alternatives to titanium implants [4,5,6]. Clinical data are available, reporting survival rates of 95.4% after 3 years [4] and 98.4% after 5 years in situ [5].

Permanent osseointegration, indicated by the formation of a direct bone–implant contact, is the most important requirement for the clinical success of an implant [7]. The endosseous part of the implant is shaped as screw to achieve a certain primary stability after insertion. Additionally, most implant surfaces are micro-structured, which is reported to enhance osseointegration [8]. For zirconia implants, different approaches are undertaken to structure the endosseous surface such as sandblasting, acid-etching, laser structuring, additive sintering or injection molding [9,10,11]. The currently available surfaces providing long-term clinical data for zirconia implants are sandblasted followed by acid etching [4,12] and, optionally, heat treated [5].

Another approach is the creation of a biologically active implant surface by applying an additional functional layer, which has been done for titanium surfaces [13,14]. Nitrogen-rich surface chemistry is known to promote cellular attachment because it contains polar groups [15]. The most common plasma precursors used to generate amine functionalities on biomaterials are allylamine [16,17,18], ethylendiamine [19], cyclopropylamine [20,21] as well as mixtures of hydrocarbon-containing gases and molecular nitrogen [22]. Due to the presence of positively charged carriers such as NH_2_ groups on the surface coating [23,24], the net negative charged eukaryotic cells are attracted. For instance, plasma-polymerized allylamine (PPAAm) coatings have been applied on titanium [25,26,27,28,29,30], titanium alloy (Ti6Al4V) [22], porous calcium phosphate [31] and yttria-stabilized zirconia (Y-TZP) [32] to improve their hydrophilic properties by generating positively charged amine groups. The resulting zeta potential changed from negative into positive values, e.g., untreated titanium: −82.3 mV and PPAAm-coated Ti: +8.6 mV (pH 7.4) [33]. On all tested materials, the cell spreading of human osteoblastic cells MG-63 was accelerated by the PPAAm coating. PPAAm-coated titanium plates have also been inserted in the muscular neck tissue of rats, revealing lower macrophage-related reactions in the mid (14 d) and late (56 d) phases of the study than uncoated titanium specimens [29]. However, the PPAAm coating is susceptible to aging. Within 7 days after coating, 70% of the primary amino groups of the PPAAm layer were already converted into amides. Zeta potential remained positive and even increased with prolonged storage of 200 d from 13.9 ± 1.2 mV to 26.3 ± 0.5 mV (pH 6.0), probably due to the increased density of imines, nitriles and acid amides [30]. Nevertheless, the cell spreading of human osteoblasts on PPAAm-coated titanium alloys that were stored over 360 d was accelerated compared to uncoated specimens [30]. To evaluate the differentiation behavior of osteoblastic cells, gene expression of differentiation markers such as alkaline phosphatase (ALP), collagen type 1 (COL) or osteocalcin (OCN) are measured. COL and ALP are considered early differentiation markers in the osteoblast lineage, while the transcription of OCN is enhanced in a later differentiation stage. The purpose of the present study is to test whether a PPAAm coating on zirconia is stable up to 5 years, which is the common shelf life of ready-for-sale implants. The reaction of human osteoblasts to the PPAAm coating on zirconia has therefore been assessed by evaluating cell viability, spreading, cell morphology and gene expression.

## 2. Materials and Methods

Zirconia discs with a diameter of 13 mm and a height of 2 mm were produced. The discs were machine overdimensioned in the green state, sintered and isostatically hot pressed in order to get disc-shaped specimens. The zirconia was composed of 93.0 wt% ZrO_2_, 5.0 wt% Y_2_O_3_, 0.1 wt% Al_2_O_3_, 1.9 wt% HfO_2_; its grain size was 0.3 µm (MZ111, CeramTec, Plochingen, Germany). Four different surfaces were produced according to Table 1: ZA0: as-sintered zirconia, heat treated for 1 h at 1250 °C, PPAAm coating, ZA5: as-sintered zirconia, heat treated for 1 h at 1250 °C, PPAAm coating, aged 5 years in sealed package, Zmt: as-sintered zirconia, heat treated for 1 h at 1250 °C, Z14: sandblasted Al_2_O_3_ 105 µm, etched for 1 h in hydrofluoric acid 38–40%, heat treated for 1 h at 1250 °C. Z14 is the endosseous surface of a clinically tested implant [5] (cer.face 14, Vita, Bad Säckingen, Germany) and served as the clinically relevant control. Z14 displayed the following roughness parameters: arithmetical mean (Ra) = 1.47 ± 0.0.6 µm, maximum height of profile (Rz) = 10.85 ± 0.67 µm [34]. Specimens were heat treated at 1250 °C for 1 h to achieve a higher tetragonal phase of zirconia and consequently increase its resistance to aging. The surface of Zmt (Ra = 0.33 ± 0.0.2 µm, Rz = 2.71 ± 0.24 µm [34]) served as a substrate for the specimens treated with PPAAm (ZA0 and ZA5). Those specimens were coated with a thin (approximately 40 nm) PPAAm layer using a low-pressure plasma reactor (V55G, Plasma Finish, Germany) according to the following two-step procedure: (1) activation of the substrates by a continuous wave oxygen/argon plasma (500 W, 50 Pa, 1000-sccm O2/5 sccm Ar) for 60 s and (2) deposition of PPAAm by microwave-excited (2.45 GHz) pulsed plasma (500 W, 50 Pa, 50 sccm Ar) for 480 s (effective treatment time). Prior to flushing the reactor with allylamine, the precursor was carefully purified of air by evacuating and purging with N_2_. Substrates were treated in a downstream position 9 cm from the microwave coupling window. ZA0 and ZA5 were then immediately stored in aseptic packaging until use. Specimens of ZA5 were stored in aseptic packaging at room temperature for a period of 5 years. Prior to all experiments, Z14 and Zmt specimens were cleaned in an ultrasonic bath, 70% ethanol for 5 min, distilled water for 5 min, sterilized in a heating chamber at 200 °C for 2 h (FED-240, Binder, Tuttlingen, Germany) and stored in sterile petri dishes that were wrapped with aluminum foil for at least 2 weeks. The specimen surfaces were then characterized in terms of their elemental composition and visualized using scanning electron microscopy (SEM).

### 2.1. Specimen Characterization

#### 2.1.1. Elemental Composition (XPS)

The elemental surface composition was analyzed by high-resolution scanning XPS. The spectra were acquired using an Axis Supra delay-line detector (DLD) electron spectrometer (Kratos Analytical, Manchester, UK) equipped with a monochromatic Al K_α_ source (1486.6 eV). The analysis area was approximately 250 µm in diameter during the acquisition, obtained by using the medium magnification lens mode (field of view 2) and by selecting the slot mode. The core level spectra of each element, which were identified in the survey spectra, were collected at a pass energy of 80 eV by applying an emission current of 10 mA and a high voltage of 15 kV. Charge neutralization was implemented by a low-energy electron injected into the magnetic field of the lens from a filament located directly atop the sample. For each sample, spectra were recorded on three different spots and randomly distributed. Data processing was carried out using CasaXPS software, version 2.3.22PR1.0 (Casa Software Ltd., Teighnmouth, UK). Due to sample charging, the binding energy scale was corrected for all samples by setting the carbon C1s binding energy to 285.0 eV. Concentrations are provided in atomic percent (at%). The labeling of primary amino groups was performed with 4-trifluoromethyl-benzaldehyde (TFBA, Alfa Aesar, Haverhill, MA, USA) at 40 °C in a saturated gas phase for 2 h. The density of the amino groups, the ratio of NH_2_ to carbon atoms (NH_2_/C), was determined from the fluorine elemental fraction.

#### 2.1.2. SEM Imaging

The specimens’ surfaces were gold-sputtered and visualized with a scanning electron microscope (SEM) using mixed secondary electrons (SE) and backscattered electrons (BSE) modes at 15 kV (ESEM XL30, Philips, Eindhoven, the Netherlands).

### 2.2. Cell Behavior

#### 2.2.1. Cell Cultivation

The human osteoblastic cell line MG-63 (American Type Culture Collection ATCC, CRL1427) was cultivated in Dulbecco’s modified Eagle medium (DMEM + GlutaMAX-l + 4.5 g/L DGlucose + Pyruvate; gibco, Thermo Fisher Scientific, Waltham, MA, USA) with the addition of 10% fetal calf serum (FCS superior standardized S0615 0879F, Biochrom, Berlin, Germany) and 1% antibiotic (gentamicin, ratiopharm, Ulm, Germany) to 70–80% confluency up to passages 8–21 [35] at 37 °C in a humidified atmosphere with 5% CO_2_. Cells were detached with 0.05% trypsin/0.02% ethylenediaminetetraacetate (EDTA, PAA Laboratories GmbH) for 5 min at 37 °C. After stopping trypsinization by the addition of a complete cell culture medium, an aliquot of 100 μL was put into 10 mL of CASY ton buffer solution (Roche Innovatis, Reutlingen, Germany) and the cell number was measured in the counter CASY Model DT (Schärfe System, Reutlingen, Germany). Specimens were seeded with the appropriate cell number and incubated in 24-well plates (Greiner Bio-One, Frickenhausen, Germany) for the respective time intervals. All cell experiments were performed independently three times using different cell passages.

#### 2.2.2. Cell Viability

The mitochondrial dehydrogenase activity of MG-63 cells on the respective specimens was measured by 3-(4,5-dimethylthiazol-2-yl)-2,5-diphenyltetrazolium bromide (MTS) assay to determine cell viability. A drop of 120 µL cell culture medium containing 5 × 10^4^ MG-63 cells was carefully placed on each specimen (n = 2 per group) and incubated for 20 min to ensure cell attachment on the specimens. Afterwards, 1 mL of cell culture medium was added per well and the specimens were incubated for 24 h at 37 °C. Specimens were transferred to a new 24-well plate with MTS solution (CellTiter 96 ONE-Solution Cell Proliferation Assay, Promega, Madison, WI, USA) and culture medium (1:5) was added to each specimen. Blanks containing a specimen of each group with culture medium but without cells and a control group with cells growing on polystyrene were additionally tested. After 80 min, supernatants were transferred to a 96-well plate (for each specimen 3 × 80 μL were analyzed). The optical density (OD) was recorded at 490 nm with a micro-plate reader (Anthos, Mikrosysteme, Krefeld, Germany). Relative cell viability was calculated using the following equation:

Relative cell viability = (OD_specimen_ − OD_blank specimen_)/(OD_control_ − OD_blank control_)

#### 2.2.3. Cell Spreading

Cell spreading was assessed on all surfaces after 20 min and 24 h, respectively. In total, 10^6^ cells were suspended in 250 µL diluent C and their cell membranes were stained with PKH-26, a lipophilic membrane dye (PKH-26 general cell linker kit, Sigma-Aldrich, Steinheim, Germany) for 5 min at 37 °C using a dilution of 2 µL PKH-26 + 248 µL diluent C. After stopping the staining reaction using FCS, cells were washed with Dulbecco’s phosphate buffered saline (PBS), resuspended in cell culture medium and 3 × 10^4^ cells were seeded per specimen. After 20 min or 24 h, cells were rinsed twice with Dulbecco’s phosphate buffered saline (PBS) (Sigma-Aldrich), fixed with 4% paraformaldehyde for 10 min at room temperature (RT), rinsed with PBS and embedded with mounting medium (Fluoroshield with DAPI, Sigma-Aldrich) and a cover slip. Cells were examined with a water immersion objective (C Apochromat 40×, 1.2 W, Carl Zeiss, Oberkochen, Germany) at a wavelength of 546 nm using a confocal laser scanning microscope (LSM780, Carl Zeiss, Oberkochen, Germany; ZEN 2011 software black version, Carl Zeiss, Oberkochen, Germany). The mean spreading area in µm^2^ of 40 cells per specimen was then calculated using image processing software (ImageJ, v2.0.0, National Institutes of Health, Bethesda, Maryland, USA).

#### 2.2.4. Cell Morphology

The morphology of 4 × 10^4^ cells on the respective specimens after 20 min and 24 h was visualized using SEM. Cells on the specimens were rinsed with PBS after the respective time intervals, fixed with 2.5% glutaraldehyde (Merck KGaA, Darmstadt, Germany) for 30 min at 4 °C, rinsed with PBS, dehydrated with ethanol (30%, 50%, 70%, 90%, abs.), dried in a desiccator with silica gel and gold-sputtered.

#### 2.2.5. Gene Expression

On each specimen, 3 × 10^4^ cells were seeded (n = 2 per group) and cultivated for 24 h or 3 d, respectively. Total RNA was purified using the NucleoSpin RNA kit (Machery-Nagel, Düren, Germany) after the cell lysate of the 2 specimens per group was pooled. The RNA concentration for each group was measured using NanoDrop 1000 (Peqlab/VWR, Erlangen, Germany).

After isolating total RNA, first-strand cDNA was synthesized from at least 400 ng total RNA by reverse transcription with SuperScript II (Life Technologies, Darmstadt, Germany) using 2.5-µM random hexamers (Life Technologies) (MiniCycler, MJ Research/Biozym Diagnostik, Hess, Germany). cDNA of each group, resulting from the reverse transcription, was diluted with RNase free H_2_O 1:2.5. Twelve-µL Mastermix. composed of 10-µL TaqMan Universal PCR Master Mix (Life Technologies), 1-µL RNase free H_2_O and 1-µL Assays-on-Demand gene expression assay mix (Life Technologies) for the detection of either alkaline phosphatase (ALP, #Hs00758162_m1ALPL), collagen type 1 (COL I, #Hs00164004_m1COLA1) or osteocalcin (OCN, #Hs01587813_g1BGLAP) and for glyceraldehyde 3-phosphate dehydrogenase as an endogenous control (GAPDH, #Hs99999905_m1GAPDH, housekeeping gene) was analyzed with 8 µL of cDNA.

Quantitative real-time PCR assays were performed with a 3 × 20-µL reaction mix per group, marked and monitored with the ABI PRISM 7500 sequence detection system (Applied Biosystems, Darmstadt, Germany). Relative mRNA expression for each marker protein was calculated based on the comparative ΔΔCT-method, normalized to GAPDH as an endogenous control and calibrated to the control cells grown on polystyrene after 24 h.

### 2.3. Statistical Analysis

Data are presented as the mean and its standard deviation. Values were analyzed for normal distribution using the Shapiro–Wilk test. For normally distributed data, one-way ANOVA was applied followed by a post-hoc Fisher least significant difference (LSD) test to determine differences between groups. Values of gene expression were analyzed with Student’s *t*-test. The level of significance was set to α = 0.05.

## 3. Results

### 3.1. Specimen Characterization

The elemental surface compositions of the coated samples, ZA0 and ZA5, determined with XPS, are listed in Table 2. The PPAAm coating on ZA0 is mainly composed of carbon and nitrogen, which were the constituents of the precursor used for the plasma polymerization (except for hydrogen, which cannot be analyzed by XPS), as well as a marginal fraction of oxygen that originates from post-oxidation processes. For the aged PPAAm coating (ZA5), a remarkably higher portion of oxygen was determined compared to ZA0 and, furthermore, traces of zirconium, silicon, fluorine and chloride at a total amount of <1 at% were detected. The amino group density of NH_2_/C was found to be 3.4% for the as-deposited PPAAm coating (ZA0) and 0.3% for the 5-year aged layer (ZA5).

**Figure 1:**
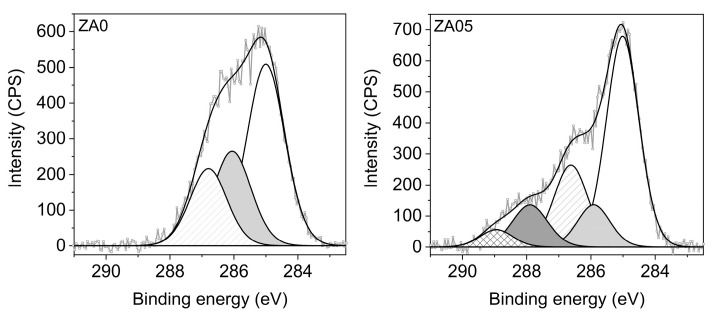
XPS C1s high resolution spectra of PPAAm after preparation (ZA0) and aging in a sealed aseptic packing at ambient conditions for 5 years (ZA5).

The high-resolution XPS C1s spectra of ZA0 and ZA5 are shown in Figure 1. The PPAAm C1s peak of ZA0 can be fitted with three components: one at 285.0 eV, characteristic for C−H or/and C−C aliphatic bonds, another at 285.9 eV assigned to C−NH, and a third component at 286.8 eV, which corresponds to C–O, C–O–C, C = N or nitriles. In contrast, the highly resolved C1s spectrum of ZA5 shows drastic changes in the shape of the C1s peak with two further components at 287.9 eV and 289.0 eV attributed to C = O and O−C = O, respectively.

**Figure 2:**
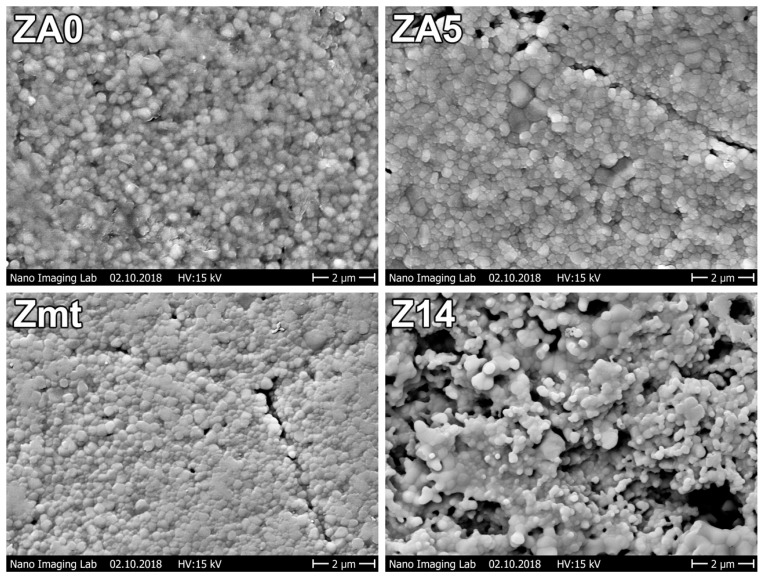
Specimen surface morphologies (SEM 10,000×).

SEM images of specimens are displayed in Figure 2. No differences between the surfaces of Zmt, ZA0 and ZA5 could be observed in SEM images. Granules can be observed on all surfaces. Z14 displayed a rougher surface with micro-rough lacunae due to sandblasting with Al_2_O_3_ particles (105 µm) and hydrofluoric acid etching.

### 3.2. Cell Behavior

**Figure 3:**
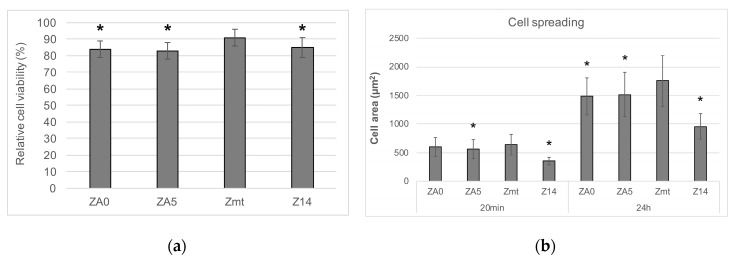
(**a**) Mean relative cell viability and standard deviation after 24 h in% normalized to the control cells grown on well bottoms. Statistically significant differences in specimens compared to uncoated zirconia specimens (Zmt), determined with a post-hoc Fisher LSD test, are indicated with * (*p* < 0.001). (**b**) Human osteoblastic cell (MG-63) area after 20 min and 24 h on differently treated zirconia specimens. Statistically significant differences in specimens compared to the control Zmt of the respective group of either 20 min or 24 h, determined with a post-hoc Fisher LSD test, are indicated with * (*p* < 0.001), n = 40 cells per group × 3 independent experiments, mean ± standard deviations.

Cell viability after 24 h was significantly higher for Zmt than for all other specimens (*p* < 0.001) (Figure 3a). Cell spreading after 20 min was significantly highest for Zmt = ZA0 > ZA5 > Z14, while, after 24 h, spreading was also significantly different between Zmt > ZA0 > ZA5 > Z14 (*p* < 0.001) (Figure 3b), possibly influenced by the increased roughness of Z14.

**Figure 4:**
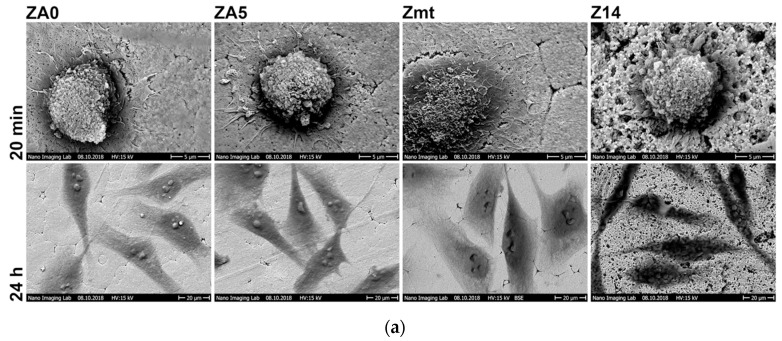

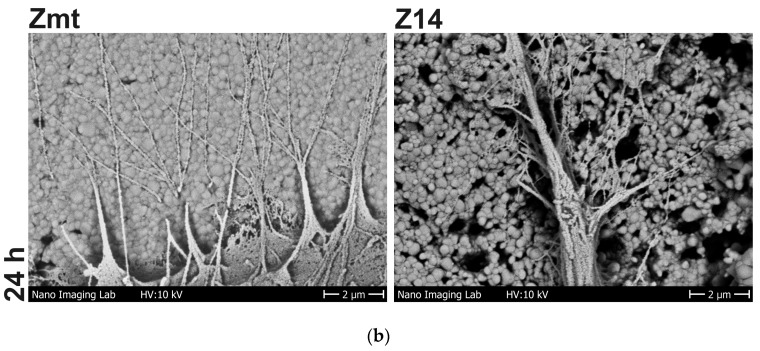
(**a**) MG-63 cells on differently treated zirconia surfaces. First row: spreading after 20 min (SEM, 5000×, bar 5 µm); second row: spreading after 24 h (SEM, 1000×, bar 20 µm). (**b**) MG-63 cell filopodia formation and interaction with the substrates Zmt and the micro-structured endosseous surface of a zirconia implant (Z14) (SEM, 10,000×, bar 2 µm).

The cell morphology visible in SEM images in Figure 4a was in accordance with the spreading determined with LSM. After 20 min, cells start to change from spherical into planar shapes; this process proceeds even further on the smooth surfaces of ZA0, ZA5 and Zmt compared to Z14. After 24 h, cells appear to be spread further on Zmt than on all other surfaces. Exposed nucleoli can be observed in the center of the cells on surfaces ZA0, ZA5 and Zmt. Due to the higher roughness on Z14, cells are less spread, but they are extended into the microstructures, where they anchor their filopodia (Figure 4b).

**Figure 5:**
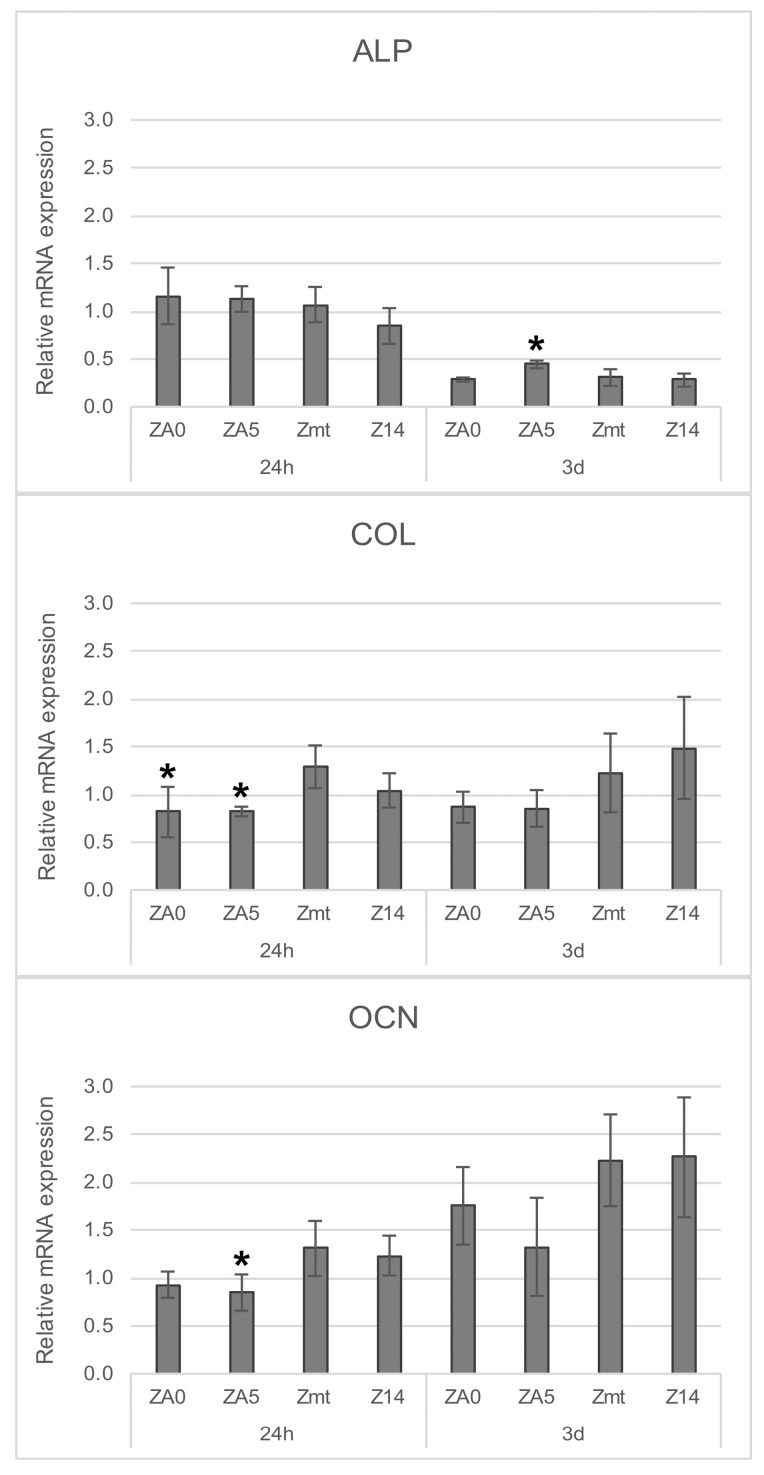
Relative mRNA expression of alkaline phosphatase (ALP), collagen type 1 (COL) and osteocalcin (OCN) in MG-63 cells on zirconia surfaces ZA0, ZA5, Zmt and Z14. The relative mRNA expression is normalized to the control cells grown on well bottoms after 24 h (=1.0), statistically significant differences in Zmt after 24 h or 3 d, respectively, determined with Student’s t-test, are indicated with * (*p* < 0.05).

The gene expression of early osteogenic marker ALP and late markers COL and OCN is displayed in Figure 5. The relative mRNA of ALP was significantly reduced on all specimens after 3 d when compared to the control cells grown on well bottoms for 24 h; COL remained stable and OCN was significantly increased for all specimens except ZA5. Significant differences when compared to Zmt at the respective time intervals are displayed in Figure 5 (*p* < 0.05).

## 4. Discussion

The purpose of the present study was to determine whether a PPAAm coating on zirconia is stable for up to 5 years and still able to improve the osteoblast reactions compared to previously reported results on titanium [25,26,27,28,29], porous calcium phosphate [31] and Y-TZP [32]. Surprisingly, the control surface of the as-sintered and heat-treated zirconia (Zmt) was the substrate that accelerated initial cell behavior. In contrast to previous findings for Y-TZP [32], in this study, a coating with PPAAm on the Zmt surface did not have an additional positive effect on the cells and even reduced their viability and spreading capability. The five-year aging of the PPAAm surfaces resulted in the oxidation of the coating, which, however, did not affect cell behavior differently than for freshly coated specimens, as also previously reported for titanium [30].

PPAAm films on ZA0 exhibited a N/C value of almost 32%—close to the theoretical N/C value of 33% for the precursor allylamine—which indicates the presence of nitrogen-containing functional groups at the surface. Additionally, XPS analysis revealed no further elements originating from the zirconia substrate (i.e., Al, Hf, Y, Zr), which confirms the homogeneous coverage of the substrate with a nanometer-thin PPAAm film. Amine-bearing plasma polymer coatings are susceptible to oxidation when stored under ambient conditions [30]. Hence, for PPAAm-coated specimen ZA5, stored for 5 years, the uptake of the oxygen content and a considerable depletion of the amino group density were determined by XPS.

The SEM images of the specimens revealed a surface structure with rounded granules sized around 100 nm. Due to the sandblasting and etching with hydrofluoric acid of Z14, niches were formed and surface roughness consequently increased. Since the thickness of the PPAAm layer is around 40 nm, the surface textures of ZA0 and ZA5 are comparable to the control Zmt.

Cell viability was significantly highest for Zmt than for all other surfaces. It has been previously seen that viability on smooth surfaces is increased compared to micro-structured surfaces [34]. The presence of the PPAAm layer also reduced cell viability when compared to the control without a coating. However, the viability of cells on the PPAAm-coated surfaces was comparable to Z14 and was above 80%; consequently, no toxic effect was initiated by the PPAAm coating when considering ISO standard 10993-5, which indicates no toxic effects for cell viability > 75%. The biocompatibility of a PPAAm coating on titanium has previously been tested in a rat model and no increased local inflammation compared to uncoated specimens was observed [29].

The initial cell spreading of MG-63 osteoblasts has been identified as the main factor that is highly accelerated by the PPAAm coating on titanium surfaces [27,30,31]. However, spreading on zirconia could not be improved when the coating was applied in the present study and was even lower than on uncoated specimens after 20 min as well as after 24 h. In contrast to the present study using 10% FCS, previously, no serum was added to the cell culture medium when cells were seeded on the PPAAm-coated specimens. Serum proteins improve initial cell adhesion and the spreading of osteoblasts on zirconia [36] and the addition of fetal calf serum to the culture medium may have masked the potential effects of PPAAm. That spreading was generally higher on smooth than on micro-roughened surfaces has been previously observed for osteoblasts on zirconia [37,38], as well as on titanium [39]. Adequate spreading is a crucial factor for the proliferation of adherent cells because maximum extracellular matrix contact with the whole cell body is aspired to maintain osteoblastic function [40,41].

The cell morphology visible in SEM images was in accordance with the measured cell areas in LSM images. On Z14 surfaces, cells anchored their filopodia on the micro-roughened surface and spread into the depths of the niches; hence, the cell area appeared smaller than for cells on all other surfaces, as previously seen for primary human osteoblasts (HOB) [38].

For the gene expression of RNA markers, the downregulation of ALP after 3 d, stable COL and upregulated OCN in MG-63 cells, as observed, can be considered typical reactions in osteoblast maturation. COL and ALP are early differentiation markers in the osteoblast lineage; hence, mRNA expression of these markers is increased in preosteoblasts and declines during osteoblast maturation [42]. mRNA expression of COL was significantly increased for cells on Zmt when compared to PPAAm-coated specimens after 24 h. OCN is first expressed at very low levels and, later in osteoblast maturation, transcription is enhanced [43]. In the present study, after 3 d, this was noticeable for ZA0 but further progressed for Zmt and Z14. In general, gene expression on Zmt and Z14 was comparable. Another study compared the gene expression of the same markers of human primary osteoblasts on machined as well as on sandblasted, etched and heat-treated zirconia [38]. ALP and COL mRNA expression of primary human osteoblasts on both zirconia surfaces was downregulated after 3 d. OCN was further upregulated for cells on the machined surface than on the sandblasted, etched and heat-treated surface after 3 d [38]. These gene expression results support the finding of the present study that smoother zirconia surfaces are favorable for initial cell behavior. Contrary to previous findings on titanium, calcium phosphate and Y-TZP, a coating of zirconia with PPAAm in the presence of serum in the culture medium does not improve cell behavior further. Five-year aging of PPAAm-coated zirconia resulted in the oxidation of the layer, but did not affect cell behavior differently than for freshly coated zirconia.

## 5. Conclusions

Within the limitations of this study, it can be concluded that zirconia coated with PPAAm does not additionally improve osteoblast behavior in cell culture experiments due to the high biocompatibility of zirconia.

## Figures and Tables

**Table 1:** Pretreatment of zirconia surfaces of the respective groups.

Group	Surface Pretreatment
ZA0	heat treated for 1 h at 1250 °C, plasma-polymerized allylamine coating September 2018
ZA5	heat treated for 1 h at 1250 °C, plasma-polymerized allylamine coating August 2013
Zmt	heat treated for 1 h at 1250 °C
Z14	sandblasted Al_2_O_3_ 105 µm, etched 1 h hydrofluoric acid 38–40%, heat treated for 1 h at 1250 °C

**Table 2:** Elemental composition of plasma-polymerized allylamine (PPAAm)-coated zirconia surfaces (ZA0) and aged surfaces (ZA5) determined with XPS.

	C (at.%)	N (at.%)	O (at.%)	N/C (%)	O/C (%)	NH_2_/C (%)
**ZA0**	74.5 ± 2.1	22.9 ± 2.3	2.6 ± 0.3	30.7 ± 3.8	3.5 ± 0.3	3.4 ± 0.1
**ZA5**	73.9 ± 0.8	13.9 ± 0.8	11.4 ± 0.3	18.8 ± 1.3	15.4 ± 0.1	0.3 ± 0.1
